# MR imaging profile and histopathological characteristics of tumour vasculature, cell density and proliferation rate define two distinct growth patterns of human brain metastases from lung cancer

**DOI:** 10.1007/s00234-022-03060-2

**Published:** 2022-10-03

**Authors:** Makoto Kiyose, Eva Herrmann, Jenny Roesler, Pia S. Zeiner, Joachim P. Steinbach, Marie-Therese Forster, Karl H. Plate, Marcus Czabanka, Thomas J. Vogl, Elke Hattingen, Michel Mittelbronn, Stella Breuer, Patrick N. Harter, Simon Bernatz

**Affiliations:** 1 Institute of Neuroradiology, University Hospital, Goethe University, Frankfurt am Main, Germany; 2 Department of Neurology, University Hospital, Frankfurt am Main, Germany; 3 Frankfurt Cancer Institute (FCI), Goethe University, Frankfurt am Main, Germany; 4 University Cancer Center Frankfurt (UCT), University Hospital, Goethe University, 60590 Frankfurt am Main, Germany; 5 Institute for Biostatistics and Mathematical Modelling, University Hospital, Frankfurt am Main, Germany; 6 Neurological Institute (Edinger Institute), University Hospital, Frankfurt, Frankfurt am Main, Germany; 7 Senckenberg Institute of Neurooncology, University Hospital, Frankfurt am Main, Germany; 8 German Cancer Consortium (DKTK), Heidelberg, Germany; 9 German Cancer Research Centre (DKFZ), Heidelberg, Germany; 10 Department of Neurosurgery, Goethe University, Frankfurt am Main, Germany; 11 Department of Diagnostic and Interventional Radiology, University Hospital Frankfurt, Goethe University Frankfurt Am Main, Theodor-Stern-Kai 7, 60590 Frankfurt am Main, Germany; 12 Luxembourg Centre for Systems Biomedicine (LCSB), University of Luxembourg, Esch-sur-Alzette, Luxembourg; 13 Laboratoire National de Santé (LNS), Dudelange, Luxembourg; 14 Luxembourg Center of Neuropathology (LCNP), Dudelange, Luxembourg; 15 Department of Cancer Research (DoCR), Luxembourg Institute of Health (L.I.H.), Luxembourg, Luxembourg; 16 Department of Life Sciences and Medicine (DLSM), University of Luxembourg, Esch-sur-Alzette, Luxembourg; 17 Faculty of Science, Technology and Medicine (FSTM)S, University of Luxembourg, Esch-sur-Alzette, Luxembourg

**Keywords:** Brain metastasis, Magnetic resonance imaging, Imaging biomarker, Tumour vasculature, Histopathology

## Abstract

**Purpose:**

Non-invasive prediction of the tumour of origin giving rise to brain metastases (BMs) using MRI measurements obtained in radiological routine and elucidating the biological basis by matched histopathological analysis.

**Methods:**

Preoperative MRI and histological parameters of 95 BM patients (female, 50; mean age 59.6 ± 11.5 years) suffering from different primary tumours were retrospectively analysed. MR features were assessed by region of interest (ROI) measurements of signal intensities on unenhanced T1-, T2-, diffusion-weighted imaging and apparent diffusion coefficient (ADC) normalised to an internal reference ROI. Furthermore, we assessed BM size and oedema as well as cell density, proliferation rate, microvessel density and vessel area as histopathological parameters.

**Results:**

Applying recursive partitioning conditional inference trees, only histopathological parameters could stratify the primary tumour entities. We identified two distinct BM growth patterns depending on their proliferative status: Ki67_high_ BMs were larger (*p* = 0.02), showed less peritumoural oedema (*p* = 0.02) and showed a trend towards higher cell density (*p* = 0.05). Furthermore, Ki67_high_ BMs were associated with higher DWI signals (*p* = 0.03) and reduced ADC values (*p* = 0.004). Vessel density was strongly reduced in Ki67_high_ BM (*p* < 0.001). These features differentiated between lung cancer BM entities (*p* ≤ 0.03 for all features) with SCLCs representing predominantly the Ki67_high_ group, while NSCLCs rather matching with Ki67_low_ features.

**Conclusion:**

Interpretable and easy to obtain MRI features may not be sufficient to predict directly the primary tumour entity of BM but seem to have the potential to aid differentiating high- and low-proliferative BMs, such as SCLC and NSCLC.

## Introduction

Brain metastases (BMs) are the most common intracranial neoplasms in adults showing an increasing incidence [1–3]. BMs are usually associated with an advanced tumour stage as well as high patient morbidity and mortality. Despite complex multimodal treatment approaches including surgery, stereotactic radiosurgery or whole brain irradiation, overall survival of patients with BM still remains poor, often not exceeding 6 months [4], and only a small proportion of patients show long-term survival [4]. In patients presenting with a cancer of unknown primary and a single intracranial metastasis, magnetic resonance imaging (MRI) diagnosis of BM is particularly challenging. In case of single metastases, it might be difficult to differentiate them from malignant primary brain tumours or brain abscess [5, 6]. Some studies described hypointense T2-weigthed imaging signal in adenocarcinomas and investigated related histopathological features [7, 8]. Previous histopathological and MR neuroimaging studies investigated the prognostic value of single parameters such as brain oedema [9, 10].

Signal intensities or lesion pattern on conventional MRI or other MRI parameters such as water diffusion may aid in the characterisation of diseased brain tissue. Reduced apparent diffusion coefficient (ADC) in brain tumours, calculated from diffusion-weighted imaging (DWI) [11], is found in areas with higher cell density, since the increased number of cells narrow the extracellular space [12–14]. Consequently, DWI parameters correlate with histopathological characteristics of primary brain tumours, such as tumour entity, tumour grading [15] and Ki67 tumour cell proliferation index [16]. However, it is unclear if DWI and other MRI patterns may have the potential to allow for the differentiation of the BM primary tumour entity. Such a differentiation would ease the diagnostic workup. For example, inconclusive biopsies of lung cancers may implicate surgery of BM that however would not be the first choice in small-cell lung cancer (SCLC), since BMs of SCLC are predominantly treated by radiotherapy. In contrast, surgical resection of the primary lung cancer and solitary BM of non-small-cell lung cancer (NSCLC) is warranted (for review, see Goldberg et al. [17]).

In this study, we aimed to elucidate discriminators for BMs originating from different primary tumours by analysing their morphological MRI features, ADC values and signal intensities on DWI, T2-weighted and T1-weighted MR images. We correlated these MRI features with histopathological parameters to validate tumour biology and to elucidate the underlying mechanism.

## Materials and methods

### Patient cohort and clinical data

We retrospectively analysed 95 preoperative MRIs with histologically proven BM (female, 50; male, 45; mean age 59.6 ± 11.5 years) from malignant melanoma (*n* = 10), breast cancer (*n* = 18), NSCLC (*n* = 30), SCLC (*n* = 8), renal cell carcinoma (*n* = 7), colon cancer (*n* = 7), carcinomas not otherwise specified (NOS) (*n* = 10) or others (*n* = 5, including rare entities like ovary carcinoma and oesophageal carcinoma). All patients underwent surgical BM resection. Preoperative Karnofsky Performance Scale (KPS) was assessed [18]. Median KPS was 80%, ranging from 20 to 100%.

### MR imaging

MRI was performed in clinical routine at different institutions (in-house *n* = 53; other institutions *n* = 42) with different field strengths (1 Tesla *n* = 4; 1.5 Tesla *n* = 57; 3 Tesla *n* = 34) and manufacturers (Siemens Healthineers *n* = 43, Philips Healthcare *n* = 42, General Electric Healthcare *n* = 10), different slice thicknesses and gaps. The in-house and external MRI protocols included at least T1-weighted images with and without contrast enhancement and T2-weighted images. Further, DWI sequences were available in 79 MRIs with ADC parameter maps in 64 cases. T2*-weighted and fluid attenuated inversion recovery sequences in 61 and 86 cases. Protocols, as well as contrast agent application, varied due to interinstitutional standardised operating procedures. We aimed to develop an approach that is feasible in clinical routine facing highly heterogenous data.

### MRI analysis

#### Assessment of peritumoural oedema

**Fig. 1:**
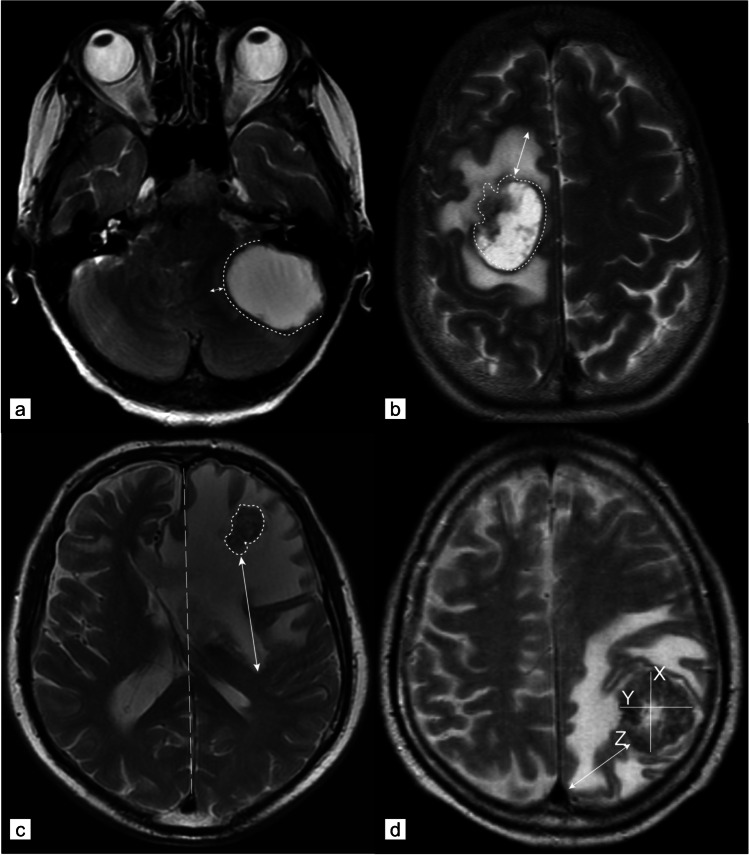
Oedema scoring. Overview of oedema scoring according to Spanberger et al. [9] and Tung et al. [19]. **a** T2-weighted MRI of a left-sided infratentorial brain metastasis with a very small peritumoural oedema (arrowed line) according to Spanberger grade 1 (maximal width 1 cm). **b** T2-weighted MRI of a right frontal metastasis with a moderate peritumoural oedema rim (arrowed line) according to Spanberger grade 2 (maximal width > 1 cm, but not crossing the midline of the brain). **c** T2-weighted MRI of a left frontal metastasis with a large peritumoural oedema (arrowed line) according to Spanberger grade 3 (maximal width > 1 cm rim and crossing the midline of the brain). **d** T2-weighted MRI with a left parietal metastasis. Example of measurements: (X) maximal orthogonal diameter, (Y) maximal horizontal diameter, (Z) maximal peritumoural oedema expansion measured from the margin of the metastasis. Peritumoural oedema ratio according to Tung et al. [19] is calculated as follows: ‘peritumoural oedema’ / (‘orthogonal diameter’ + ‘horizontal diameter’) / 2

Peritumoural oedema was defined as a region of clear T2 signal hyperintensity adjacent to the tumour margin. Measurements were performed at the maximum extent of the oedema evaluable on the T2-weighted images in axial orientation according to former studies of Spanberger et al. [9] and Tung et al. [19] (Fig. 1).

#### Region of interest (ROI) placements

For quantitative measurements, we manually drew uniform ROIs in the largest visually delineable iso- or hypointense solid tumour part of the BM by reviewing the T2- and unenhanced T1-weighted images and their corresponding structures on the DWI and ADC parameter maps yielding normalised mean values.

**Fig. 2:**
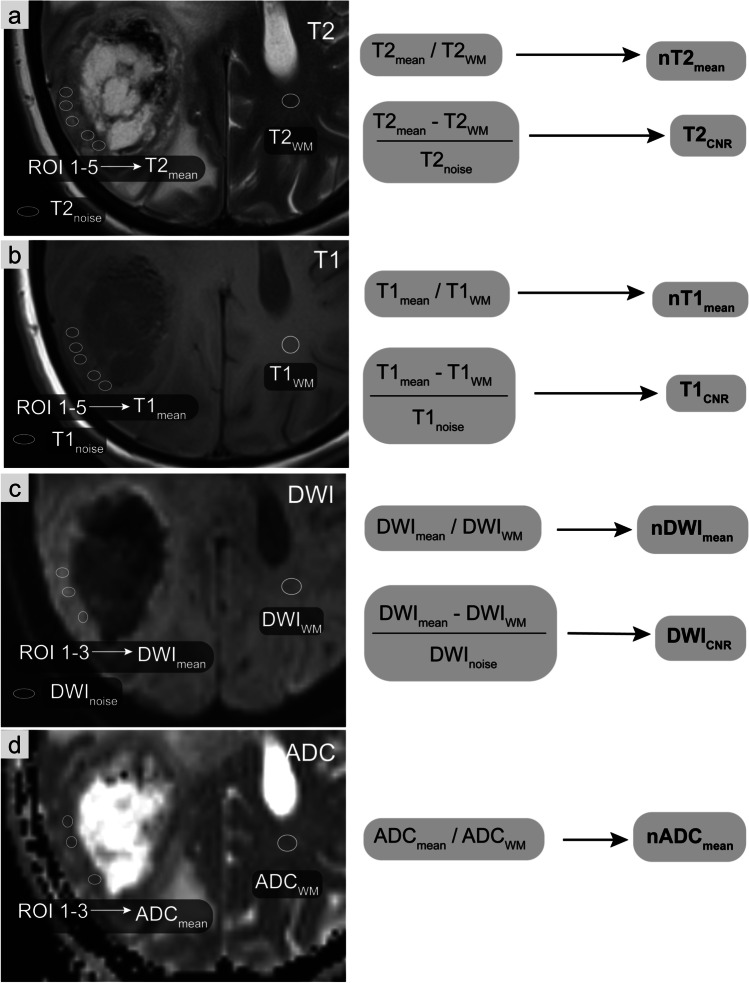
Workflow of region of interest definition and score calculation. Exemplary scheme of region of interest (ROI) placements and calculations on a right parieto-occipital brain metastasis. **a**–**b** Signal intensities of the solid tumour in the T2- (**a**) and T1- (**b**) weighted sequences were assessed by five ROIs. **c**–**d** The limited spatial resolution of the DWI (**c**) and ADC (**d**) parameter maps available allowed the assessment by no more than three ROIs. We documented the average of these ROIs (T2_mean_, T1_mean_, DWI_mean_, ADC_mean_). **a**–**d** Same-sized uniform ROIs were also drawn in the corresponding normal-appearing white matter of the contralateral hemisphere as reference values **(**T2_WM_, T1_WM_, DWI_WM_, ADC_WM_). Normalised values were established as ratios to the reference ROI yielding (nT2_mean_, nT1_mean_, nDWI_mean_, nADC_mean_). Further, we drew one ROI in the surrounding air outside the head taking its standard deviation as noise signal (T2_noise_, T1_noise_, DWI_noise_). For all ROI measurements, we always selected areas without obvious signs of bleedings (e.g. susceptibility artefacts in T2*-weighted images)

Furthermore, we calculated the contrast-to-noise ratios (T2_CNR_, T1_CNR_, DWI_CNR_) in reference to Hayashida et al. [20] by the formula: $\frac{(\mathrm T2^{\mathrm{STmean}}\;-\;\mathrm T2^{\mathrm{WM}})}{\mathrm T2^{\mathrm{noise}}}$ (only exemplarily shown for T2_CNR_). ROI placements and calculations are displayed schematically in Fig. 2.

#### Size and contrast enhancement of the BM

For assessment of BM size, we chose a slide showing the maximal extent of the lesion and measured the maximal unidimensional diameter (mm) on the axial contrast-enhanced T1-weighted images.

### Histopathological analyses

#### Tissue specimens, processing and patient data

Neuropathological examination was performed by board certified neuropathologists (KHP, MM, PNH). We investigated formalin-fixed and paraffin-embedded BM tissue obtained from the University Centre of Tumour disease Biobank Goethe University, Frankfurt am Main, Germany, member of the German Cancer consortium. All stainings were performed on tissue micro arrays. The study protocol including the usage of biomaterial was endorsed by the local ethical committee (GS 4/09; SNO-04-2015).

#### Immunohistochemistry and immunofluorescence

The tissue micro arrays were cut into 3 µm thick slices using a microtome (Leica Microsystems, Nussloch GmbH, Nussloch, Germany), placed on microscope slides (SuperFrost, Thermo Scientific, Dreieich, Germany), heated to 40 °C for 20 min (HI 1220, Leica Biosystems Nussloch GmbH, Nussloch, Germany) and stored at 37 °C overnight in an incubator (Heraeus, function line, B12, Thermo Electron Corporation, Waltham, Massachusetts, USA). Immunohistochemistry was executed using standardised protocols for the automated slide staining system Discovery XT (Roche/Ventana, Tucson, Arizona, USA) including antibodies against the following antigens: Ki67 (Dako) and CD31 (Dako). Slides were counterstained with haematoxylin and mounted. For cell density analyses, we performed a nuclear 4’,6-diamidino-2-phenylindole (DAPI) staining following deparaffinisation.

#### Data analysis

Cell proliferation was assessed by Ki67 staining and analysed using an Olympus BX-50 (Hamburg, Germany) microscope. Raw data of Ki67 proliferation rate have already been published [21] and are now included for further analyses in comparison with neuroradiological parameters. Vessel density was analysed by counting cluster of differentiation (CD) 31-positive vascular structures in relation to the analysed tumour area (per mm^2^) using a light microscope with a Stereo Investigator (Version 4.34 software from MicroBrightField Inc.). Vessel area was analysed measuring CD31-positive vessel lumina. Data analyses were performed using ImageJ 1.48v (National Institute of Mental Health, Bethesda, Maryland, USA). For cell density measurement, representative images of tumour-bearing areas of the DAPI-stained tissue micro arrays were taken using a Nikon 80i microscope (Nikon, Düsseldorf, Germany) in a 200-fold magnification with 358 nm excitation and reading of blue fluorescence in the emitting 461 nm wave length spectrum representing the nuclear staining. Intensity as a surrogate for cell density was measured using the inherent intensity measurement function of Image J.

### Statistical analysis

Comparisons between Ki67_low_ and Ki67_high_ tumours were performed using either Wilcoxon rank sum test and Pearson’s chi-square test (for ordinal scaled or non-normal distributed values) or analysis of variance in case of normal distribution (tested by Shapiro–Wilk test). For multivariate analyses, recursive partitioning conditional inference trees were build using R Software and the party package [22]. All other analyses were performed using JMP software solution (SAS, Cary, USA). For graphical illustrations, Affinity Designer (Serif (Europe) Ltd.) was used.

## Results

### Prediction of the primary cancer entity

**Fig. 3:**
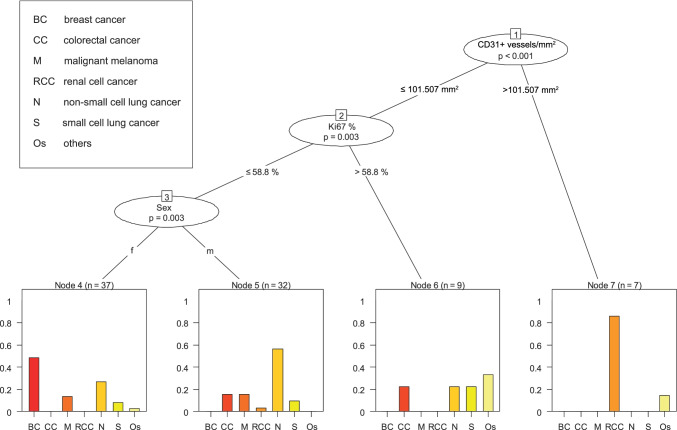
Decision tree to stratify the brain metastases’ primary tumour entity. Multivariate analysis using recursive partitioning conditional inference trees with histopathological and MRI variables as predictors of brain metastases’ primary tumour entity

Histological and clinical parameters (in particular CD31 + vessels, Ki67% and sex) had the potential to differentiate BMs’ origins (Fig. 3). We found no evidence to support our hypothesis that clinically feasible MR morphological parameters may enable the stratification of the BM’s primary tumour entity. Multivariate analysis revealed that CD31-positive vessels with a cut-off set > 101.5/mm^2^ could distinguish renal cell carcinomas from other entities (*p* ≤ 0.001). Next, BM with CD31-positive vessels ≤ 101.5/mm^2^ showed a subdivision in the proliferation rate. BMs with a Ki67 > 58.8% were diagnosed either as SCLC, ‘others’, colorectal carcinomas or NSCLC (*p* = 0.003). BM of NSCLC mainly presented with a Ki67 ≤ 58.8%. Regardless, SCLC and ‘others’ mainly appeared in the Ki67 > 58.8% group. Malignant melanomas were only found with a Ki67 ≤ 58.8% and equally distributed in gender. As expected, a differentiation of sex yielding breast cancer only appears in females (*p* = 0.003). Colorectal cancer was also found in the male cohort, with a Ki67 ≤ 58.8%. As mentioned above, NSCLC was mostly seen in Ki67 ≤ 58.8% patients, but with a predominance in males.

### Proliferation rate defines two distinct subsets of BM

After failing to predict BM’s primary tumour based on MR imaging features obtained from standard-of-care imaging, we further analysed patterns associated with the proliferative differences in BM’s.

**Fig. 4:**
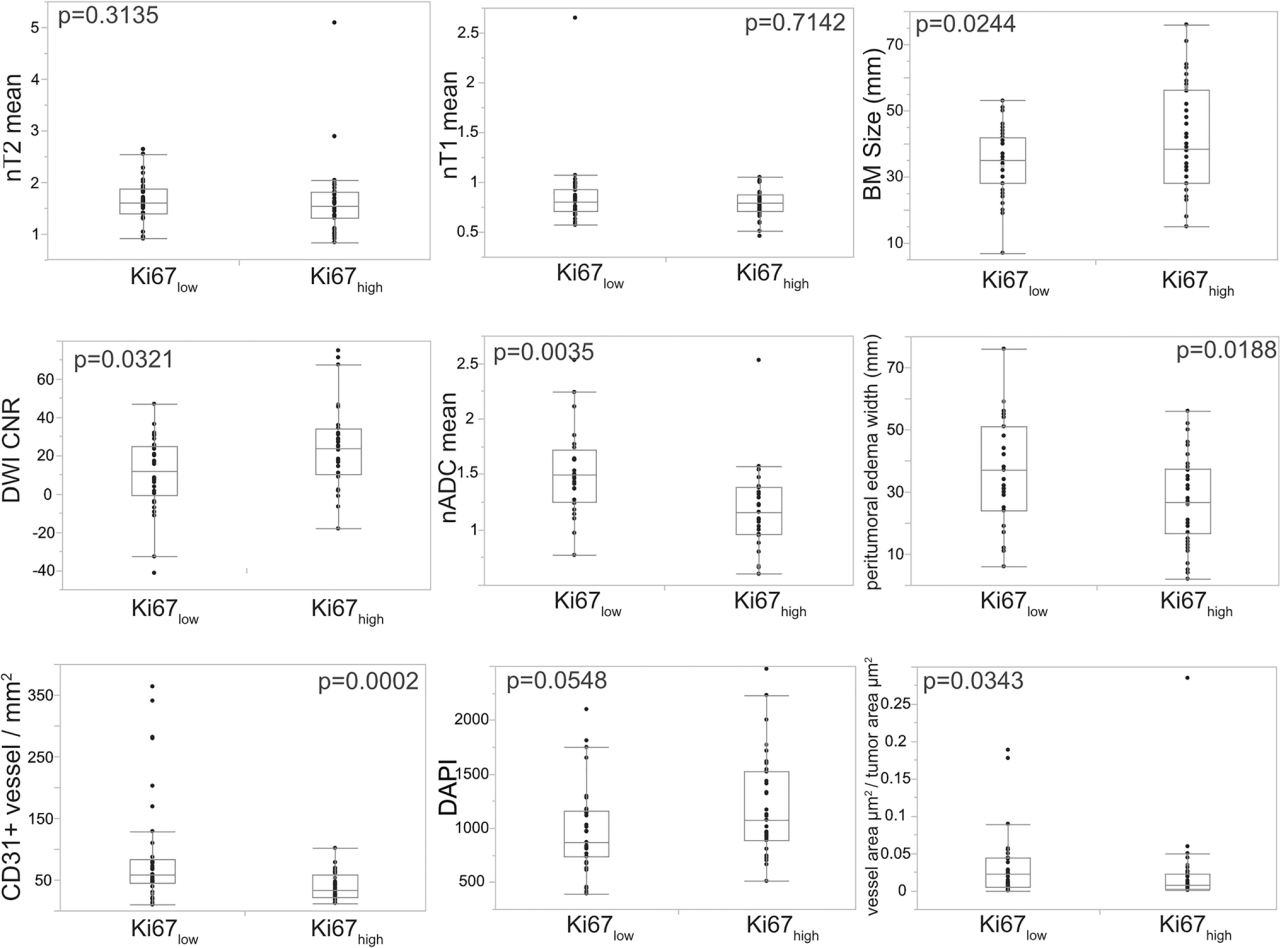
Imaging and histopathologic features differ according to the brain metastasis’ proliferation rate. Magnet resonance imaging and histopathological features in brain metastases stratified by proliferative capacity (Ki67_high_ and Ki67_low_ subgroups)

Therefore, our next step was a dichotomisation (median-split) of our BM cohort in Ki67_high_ and Ki67_low_ subgroups. BMs with a Ki67 > 27.9% and ≤ 27.9% were included in the Ki67_high_ and Ki67_low_ group, respectively. We observed that highly proliferative BM (Ki67_high_) revealed a significantly bigger tumour size (*p* = 0.0244, Fig. 4, Table 1) and a smaller peritumoural oedema (Fig. 4, Table 1) than low-proliferative BM (Ki67_low_). However, most ROI-based MR morphological parameters did not differ significantly between Ki67_high_ and Ki67_low_ BM (nT2_mean_, T2_CNR_, nT1_mean_, T1_CNR_, nDWI_mean_, Table 1). In fact, only DWI_CNR_ was significantly higher in the Ki67_high_ BM group which should represent a stronger intratumoural diffusion restriction than in Ki67_low_ BM (*p* = 0.0321, Fig. 4, Table 1). Accordingly, Ki67_high_ BM revealed lower nADC_mean_ values (*p* = 0.0035, Fig. 4, Table 1).

**Table 1:** Comparison of clinical data, magnetic resonance imaging and histological parameters of the dichotomised Ki67_high_ and Ki67_low_ brain metastases cohort

Variable		Ki67^high^ median (range) or *n* (%) or mean ± SD (CI)	Ki67^low^ median (range) or *n* (%) or mean ± SD (CI)	*p*-value
*Patient age in years (range)*		60 (33–81)	62 (13–80)	0.4206^#^
*BM number (range)*		1 (1–7)	1 (1–14)	0.9412^#^
*Sex*	Male Female	16 (21.51%) 22 (27.96%)	21 (24.73%) 18 (25.81%)	0.3026^##^
*Localization*	Supratentorial Infratentorial	21 (27.27%) 17 (22.08%)	27 (35.06%) 12 (15.58%)	0.2060^##^
*KPS (%)*		80 (20–100)	80 (30–100)	0.7471^##^
*BM size in mm (range)*		41.39 ± 15.35 (37.19–45.59)	34.58 ± 10.18 (31.29–37.89)	*0.0244^**###**^
*Peritumoural oedema according to Spanberger *et al*.* [9]	Grade 1 Grade 2 Grade 3	3 (3.90%) 15 (19.48%) 20 (25.97%)	1 (1.30%) 7 (9.09%) 31 (40.26%)	*0.0435^**##**^
*Peritumoural oedema according to Tung *et al*.* [19]	Ratio	76.37 (3.45–335.48)	112.12 (26.97–283.87)	**0.0367**^**#**^
	Width in mm (range)	26.5 (2–56)	37 (7–76)	**0.0188**^**#**^
*nT2*_*mean*_ (range)		1.55 (0.84–5.09)	1.61 (0.91–2.64)	0.3135^#^
*T2*_*CNR*_ (range)		26.23 (− 7.76–150.54)	24.70 (− 17.77–172.31)	0.9008^#^
*nT1*_*mean*_ (range)		0.79 (0.46–1.05)	0.8 (0.57–2.65)	0.7142^#^
*T1*_*CNR*_ (range)		− 20.3 (− 69.89–8.08)	− 13.48 (− 77.69–46.79)	0.2747^#^
*nDWI* ${\text{mean}}$ (range)		1.43 (0.58–2.87)	1.315 (0.63–3.22)	0.3632^#^
*DWI*_*CNR*_ (range)		23.755 (− 18.17–74.93)	11.925 (− 41.39–46.93)	**0.0321**^**#**^
*nADC*_*mean*_ (range)		1.13 (0.6–2.53)	1.48 (0.77–2.53)	**0.0035**^**#**^
$\frac{vesselarea\;{\mathrm{\mu\;m}}^{2}}{tumorarea\;{\mathrm{\mu\;m}}^{2}}$(range)		0.0079 (0.0004–0.2849)	0.0224 (0.00004–0.1886)	**0.0343**^**#**^
$\frac{CD31+vessels}{{\mathrm{mm}}^{2}}$(range)		33.19 (12.34–101.51)	59.16 (9.73–363.67)	**0.0002**^**#**^
*DAPI*		1075.56 (510.06–2473.66)	868.47 (345.78–2099.89)	0.0548^#^

*DAPI* (4′,6-diamidino-2-phenylindole). *DWI* (diffusion-weighted imaging). *ADC* (apparent diffusion coefficient). *CD* (cluster of differentiation). *CNR* (contrast-to-noise ratio). *SD* (standard deviation). *CI* (confidence interval). Age is indicated in years. KPS (Karnofsky Performance Status) is indicated in percentage (%). Brain metastasis (BM) size and peritumoural oedema width are indicated in mm. All values are labelled as median (range) or *n* (%) or mean ± standard deviation (CI). Significant *p*-values with *p* < 0.05 are indicated by *. ^#^Wilcoxon rank sum test; ^##^Pearson’s chi-square; ^###^analysis of variance was performed due to normal distribution, tested with Shapiro–Wilk test

By histopathological assessment, we found that both microvessel density (CD31-positive vessels/mm^2^) and vessel area (vessel area/tumour area) were reduced in the Ki67_high_ BM cohort (*p* = 0.0002; *p* = 0.0343, Fig. 4, Table 1). Cell density assessed by DAPI nuclear staining showed a strong trend towards a higher cell density in Ki67_high_ BM (*p* = 0.0548, Fig. 4, Table 1).

### *BM of SCLC and NSCLC as examples for Ki67*_*high*_* and Ki67*_*low*_* BM*

**Fig. 5:**
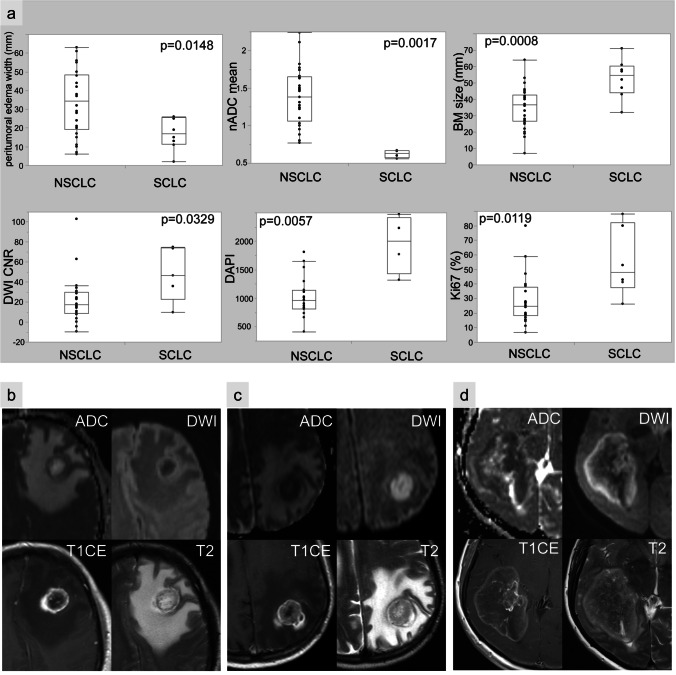
Imaging and histopathologic features differ in NSCLC versus SCLC brain metastasis. **a** Comparison of magnetic resonance imaging features and histopathological parameters in non-small-cell lung cancer (NSCLC) and small-cell lung cancer (SCLC) brain metastases. According to our findings, SCLC (**d**) usually show stronger signs of diffusion restrictions with reduced values in the ADC map, a bigger tumour size as well as a small peritumoural oedema rim compared to brain metastasis of NSCLC (**b**). Nevertheless, there are brain metastases that do not reflect these typical morphological findings, exemplified on a NSCLC brain metastasis (**c**)

As BM of NSCLC and SCLC mainly differed in their proliferative capacity and the distinction of these two lung cancer types is of major clinical relevance, we further analysed their MRI and histological parameters. BMs of SCLC (*n* = 8) were represented by the Ki67_high_ BM features, while NSCLC BM (*n* = 30) showed the pattern of the Ki67_low_ group (*p* = 0.0119, Fig. 5a). BMs of SCLC were larger (*p* = 0.0008, Fig. 5a) and showed a smaller peritumoural oedema width (*p* = 0.0148, Fig. 5a) than BM of NSCLC. Additionally, BM of SCLC showed stronger signs of diffusion restrictions by higher DWI_CNR_ signals (*p* = 0.0329, Fig. 5a) and lower nADC_mean_ values (*p* = 0.0017, Fig. 5a). On histological level, cell density (assessed by DAPI staining) was increased in BM of SCLC compared to BM of NSCLC (*p* = 0.0057, Fig. 5a).

## Discussion

In our study, we aimed to decipher MR morphological patterns of BM, to presurgically differentiate the BM’s primary tumours and to obtain biological validation by matched histological analyses.

While BMs’ MR morphology did not allow a prediction of their origin, we found different MRI features for high-proliferative (Ki67_high_) and low-proliferative (Ki67_low_) BMs that might help to distinguish BM deriving from NSCLC (Ki67_low_) and SCLC (Ki67_high_), an issue of special clinical interest facing the different treatment strategies. Therefore, the identified MR characteristics may serve as a non-invasive diagnostic tool to guide diagnostic and therapeutic decision making in patients with inoperable lung cancer and inconclusive biopsy of the primary tumour. SCLC BMs as representatives of the Ki67_high_ group were characterised by smaller surrounding oedema, despite a larger tumour size, in contrast to BMs from NSCLC reflecting the features of the Ki67_low_ group. These results seem counterintuitive at first, since an increasing peritumoural oedema has long been recognised as an important cause of morbidity and mortality in patients with metastatic lesions [9]. However, there are other examples of fast and aggressive growing brain tumours with little amounts of peritumoural oedema, for example, the group of tumours formerly known as primitive neuroectodermal tumours, atypical teratoid/rhabdoid tumours and primary central nervous system lymphomas. These highly proliferative tumours are characterised by a relatively small peritumoural oedema compared to their large tumour size [23–29]. It may be assumed that processes of vascular remodelling or less time for angiogenesis might be reasons for the limited peritumoural oedema extent of faster growing BM. It has been evaluated that along with the brain invasion of the metastatic cells, angiotrophic factors, such as vascular endothelial growth factor [30], might be released inducing the formation of new, but more leaky vessels [31]. However, as it has recently been shown for melanoma, BMs perform vascular remodelling by co-opting existing blood vessels [32, 33]. Similar to our results, Spanberger et al. [9] also found a lower mean vessel density and a lower angiogenic potential in BM with small peritumoural oedema together with a shortened patient survival. The group of Ki67_high_ BM also showed diffusion restriction with respective lower ADC values. Diffusion restriction, which means restricted diffusion of water protons in extracellular spaces, is quantified by ADC values [11]. Further, we showed that the increased diffusion restriction was associated with a trend towards an increased cellular density in Ki67_high_ BM. It has been shown before that a higher cellularity of tumours, defined as the number of cells in a given area of tumour tissue, narrows the extracellular space and limits the water diffusion [12, 13]. The extracellular water inside the tumour may also be reduced by the lower amount of permeable vasculature in Ki67_high_ BM.

In line with our findings, Berghoff et al. [10] found a correlation between the diffusion restriction and a high proliferation index (Ki67%). Furthermore, they observed a higher amount of interstitial reticulin fibres in BM presenting with signs of higher diffusion restrictions. We found no difference regarding reticulin fibres among Ki67_high_ and Ki67_low_ tumours (data not shown). Our study has limitations that warrant discussion. Due to the retrospective nature of our investigation, we faced MRIs with differing protocols, field strengths, contrast media protocols/timing and a rather small cohort size. Thus, generalisability might be limited. The sample size differed in NSCLC (*n* = 30) versus SCLC (*n* = 8) patients which might have affected the results. Larger prospective studies are warranted to disclose the relationships between the metastatic growth/proliferation and the associated structural alterations of the brain microenvironment including the neurovascular network. All ROIs were related to an internal reference and CNR was assessed whenever possible in order to allow comparability in a most standardised and objective fashion. We did not perform computerised tumour volume segmentation for the calculation of the BM size [34] and we did not calculate high-dimensional radiomics as we faced highly heterogenous real-world data and aimed to develop a clinically feasible step-by-step approach. We chose this approach since we aimed to use interpretable MR imaging features assessable in everyday clinical practice. Last, we focused on MRI measurements; therefore, the analysis of the spatial distribution of brain metastases was beyond the scope of our manuscript [35, 36].

In conclusion, we were not able to stratify a specific MRI pattern predicting BM origin. However, we detected distinct MRI features and histopathological profiles with regard to low- (Ki67_low_) and high-proliferative (Ki67_high_) BM. These features might have the potential to non-invasively differentiate NSCLC and SCLC BM in a daily routine workflow.

## Data Availability

Data is available upon reasonable request from the corresponding author.
